# Analysis of the Impact of Waste Fly Ash on Changes in the Structure and Thermal Properties of the Produced Recycled Materials Based on Polyethylene

**DOI:** 10.3390/ma17143453

**Published:** 2024-07-12

**Authors:** Renata Caban, Adam Gnatowski

**Affiliations:** 1 Department of Materials Engineering, Faculty of Production Engineering and Materials Technology, Czestochowa University of Technology, 42-201 Czestochowa, Poland; 2 Department of Technology and Automation, Faculty of Mechanical Engineering and Computer Science, Czestochowa University of Technology, 42-201 Czestochowa, Poland

**Keywords:** polymer composites, thermal properties, polyethylene, fly ash, crystallinity, structure

## Abstract

This paper presents the results of the research on the structure and thermal properties of materials made from fly ash based on high-density polyethylene (HDPE). Composites based on a polyethylene matrix with 5, 10, and 15 wt% fly ash from hard coal combustion content were examined. Fourier transform infrared spectroscopy with attenuated total reflectance (FTIR-ATR) was used to identify characteristic functional groups present in the chemical structure of polyethylene and the composites based on its matrix. Structural analysis was performed using X-ray diffraction (XRD), a differential scanning calorimeter (DSC), and microscopic examinations. Mechanical properties were also examined. Analysis of the thermal effect values determined by the DSC technique, XRD, and FTIR-ATR allowed the evaluation of the crystallinity of the tested materials. Polyethylene is generally considered to be a two-phase system consisting of crystalline and amorphous regions and is a plastic characterized by a significant crystalline phase content. Based on the FTIR-ATR spectra, DSC curves, and XRD, the effect of the filler and the changes occurring in the materials studied resulted in a decrease in the degree of crystallinity and a change in the melting point and crystallization temperature of the polymer matrix were established. Microscopic examinations were carried out to analyze the microstructure of the composites to collect information on the distribution and shape of the filler particles, indicating their size and distribution in the polymer matrix. Furthermore, the use of scanning electron microscopy combined with energy-dispersive X-ray spectroscopy (SEM-EDS) allowed for the microanalysis of the chemical composition of the filler particles.

## 1. Introduction

Fly ash is one of the combustion products that is a waste product obtained from the combustion of energy resources, such as hard coal, brown coal, and biomass, in thermal power plants [1]. Fly ash is obtained by dust capture from the emitted flue gases through dust extraction devices that precede the combustion chamber. Separated fly ash is divided into categories depending on the type of fuel from which it is derived and its physicochemical properties [2]. Further subdivisions arise from the design of the furnace, the method of ash collection from the individual electrostatic precipitators, and the technology of desulphurization of the resulting flue gases. EN 450-1:2009 [3] introduced further possibilities for classifying fly ash. The first relates to the loss of ignition, while the second divides the ashes according to their fineness, i.e., the size of the individual particles. Such an extensive subdivision allows the particles to be recycled or stored. The loss of ignition from coal that does not burn completely directly affects the color of the fly ash [4]. For many years, the country’s standards allowed the use of fly ash containing 5 to 7% carbon and was mainly used as an additive in concrete production. Fly ash containing higher carbon contents was treated as harmful waste and posed a serious threat to the environment. However, its classification has changed, and consequently, fly ash is now considered an aluminosilicate material. Such a change has contributed to the search for new ash management options. The diversity of properties resulting from the different types of initial material and the type of production process directly influences the opportunities for fly ash management. The fly ash management methods encountered to date have focused on such fields as road construction [5], mining [6], ceramics [7], agriculture [8], flood protection [9], and zeolite production [10]. A potential and also promising application of fly ash particles is their use as a lightweight filler in polymer matrices, making them viable from a technical and economic perspective. There are many publications in the literature on the use of fly ash as a filler in polymer composites [11,12,13,14]. The use of fly ash to modify polyethylene leads to obtaining a composite material with new application opportunities [15,16]. The impact of waste on the environment is of great importance, and fly ash, a type of solid waste, is the subject of research by many authors; thus, addressing the recycling of these materials and the production of innovative materials, ranging from biomass fly ash and high-density polyethylene (HDPE) to geotechnical applications, is of great importance [17]. The authors of the paper [18] produced a composite material from two industrial wastes: recycled polyethylene terephthalate and fly ash as a filler. Composites were made using a single-threaded bar’s barrel extruder. The effect of various fly ash additives on the thermal properties and microstructure of the composite material was evaluated. Thermal and microscopic analyses of the composite materials were carried out. It was observed that after the addition of fly ash, the thermal properties of the resulting composite improved, which was confirmed by tests using differential scanning calorimetry. The innovative aspect of the article includes both the method of producing injection-molded composites with optimal parameters, allowing their application in various industries, and the prediction of thermal processes for HDPE composites with fly ash from the combustion of hard coal, which is one of the most commonly used fossil fuels in the power industry. The addition of fly ash particles to a thermoplastic matrix presents many challenges when understanding the physical, thermal, and morphological changes in the composite. To develop a commercial composite material, it is necessary to analyze the effect of fly ash particles on properties and microstructure. The main objective of this study is to evaluate the use of fly ash as a cost-effective filler in polymers. The physical modification of high-density polyethylene used includes a change in structure due to forced orientation, the mixing of the polymer with the modifier, a change in the degree of dispersion and supramolecular structure of the individual phases, and a change in the structure and nature of intermolecular interactions at the interface as a result of the addition of the modifier. The use of a modifier in the form of fly ash significantly affects changes in thermal properties, crystallinity, and structure, which affects the mechanical properties of the produced polyethylene matrix composites. High-density polyethylene (HDPE) was selected as the base polymer. It is a polyolefin thermoplastic with a high degree of crystallinity. The material is used in the construction industry and for components in the automotive sector. Various types of household items, such as cups, buckets, etc., are made of HDPE. Polyethylene can also be found in beverage bottles, containers for chemicals, and components of decorative items. A very important application of high-density polyethylene is its use as a material for gas pipes.

This paper presents the results of a study on the effect of the addition of fly ash from the combustion of hard coal on the structure and crystallinity of specimens made of high-density polyethylene (HDPE). The research included X-ray examinations, FTIR-ATR spectroscopic structure analysis, thermal analysis (DSC), and microscopic examinations. To analyze the mechanical properties, a three-point bending test and hardness measurements were carried out.

## 2. Materials and Experimental Procedure

### 2.1. Materials

**Figure 1:**
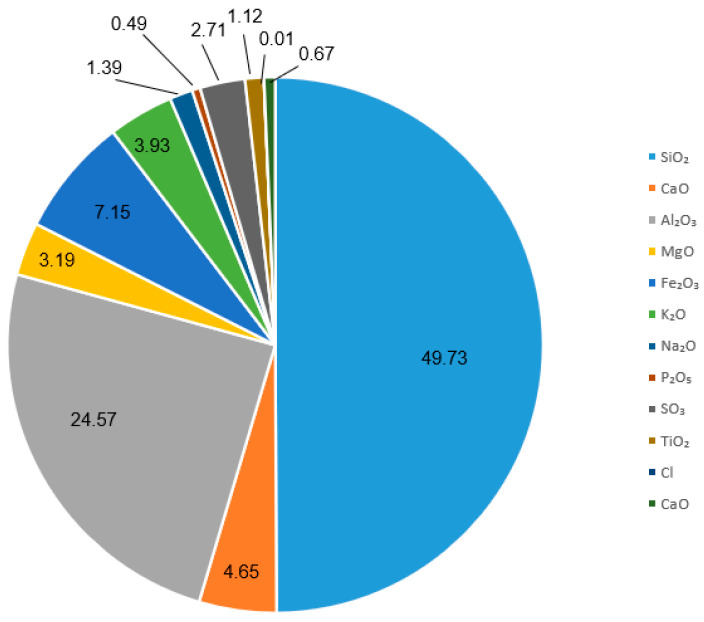
Chemical composition of fly ash from hard coal combustion.

This paper presents the results of the examinations of high-density polyethylene composites modified with fly ash from the combustion of hard coal in pulverized-fuel boilers. High-density polyethylene (HDPE) with a trade name Hostalen GC 7260 (Lyondell Basell Industries Holdings) (Houston, TX, USA) was used as a composite matrix. Fly ash (a product of hard coal combustion in Energia Polska S.A. (Warsaw, Poland) was used as a filler. The chemical composition of the fly ash is shown in Figure 1.

Before processing, polyethylene was dried in a Shini SG-2417-CE dryer (Shini Plastics Technologies, Inc., New Taipei, Taiwan) at 70 °C for 4 h. The fly ash was coated with an oil formulation applied to increase the adhesion of the filler to the polymer. Oil produced from rapeseed, subjected to mechanical cold pressing (at temperatures up to 50 °C), was used. The main component of rapeseed oil is fat, made up of monounsaturated fatty acids (63.3%) and polyunsaturated fatty acids (28.1%). Only a small part is made up of saturated fatty acids (7.4%). The energy value of rapeseed oil is 884 kcal/100 g. The test specimens (in the form of tensile samples) were obtained using injection molding technology [19] on a Krauss Maffei KM65-160C1 (KraussMaffei Group GmbH, Munisch, Germany) injection molding machine. The specimens contained 5 wt%, 10 wt%, and 15 wt% fly ash from the combustion of hard coal. Pure high-density polyethylene specimens were also produced for comparison purposes. The parameters of the injection molding process of the test specimens were as follows: maximum allowable pressure in the plasticizing system 70 MPa, an injection time of 0.7 s, clamping pressure of 35 MPa, pressing time of 25 s, cooling time of 20 s, dispensing time of 7 s, mold closing force of 650 kN, mold temperature of 50 °C, and temperatures of the cylinder zones T_1_ = 160 °C, T_2_ = 175 °C, T_3_ = 180 °C, T_4_ = 195 °C and nozzle temperature T_5_ = 190 °C. Table 1 shows the weight proportions of fly ash added to HDPE and the test sample determinations used for the samples in the following work.

### 2.2. Test Methods

#### 2.2.1. X-ray Diffraction (XRD)

X-ray diffraction (XRD) is an important technique for identifying partially crystalline polymers. An analysis of the position of diffraction peaks, intensity, width of the peak, and the shape of the XRD curve provides valuable information about the structure of the polymer material. X-ray analysis (XRD) was used to determine structural changes, crystallinity, and the size of crystallites in the materials studied. A Seifert 3003 T-T (Rich. Seifert&CO, Ahrensburg, Germany) X-ray diffractometer with a cobalt anode (λ = 0.178901 nm) was used for the analysis. The operating parameters of the lamp were as follows: U = 40 kV and I = 30 mA. Measurements were carried out for the scattering angle 2θ, ranging from 10° to 50°, with a measurement step of 0.1°.

#### 2.2.2. FTIR-ATR Studies

The examinations were performed using the ATR (attenuated total reflectance) technique. A SHIMADZU Irraffinity-1s (Kyoto, Japan) spectrophotometer with a reflection type ATR attachment, with a diamond crystal used to record the spectra. Spectroscopy research samples were cut out of the strength samples. They were in the form of cuboids with measurements of 2 × 2 × 1 mm. Sample surfaces were flat, smooth, and tightly adhered to the crystal’s surface. FTIR-ATR measurements were performed in the measurement range from 400 to 4000 cm^−1^. Sixty scans per measurement were taken, with a resolution capacity of 1 cm^−1^. The ATR spectra were subjected to baseline correction, ATR correction, and scale-based normalization operations, making the spectra equivalent to transmission spectra.

#### 2.2.3. Differential Scanning Calorimetry (DSC)

DSC examinations were carried out using a Netzsch (Netzsch Group, Selb, Germany) DSC 200 PC Phox instrument. The tests were conducted in a nitrogen atmosphere at a scan rate of 20 °C/min^−1^. The specimens for DSC testing were cut perpendicular to the direction of plastic flow in the mold from the specimens obtained by injection molding. This minimized the skin-core effect. Indium was used as a model, with the sample weight ranging from 8 to 10 mg. The test samples were weighed with a SARTORIUS balance with a weighing accuracy of 0.01 mg, internal calibration, and a closed measuring chamber. Specimens were heated from 25 °C to 180 °C (1 heating cycle) and held at this temperature for 4 minutes to eliminate the thermal history of the material. The specimens were then cooled to 25 °C. After crystallization, the specimens were reheated to 180 °C. The melting point (T_peak_) and crystallinity of the composites were determined for the maximum area of the endothermic reflex recorded in the second heating cycle. Analysis of the DSC thermograms was carried out using the Netzsch Proteus 6.1.0 software. The percentage of the crystalline phase was calculated using the following relationship:

(1) $$S_{k}=\frac{{∆H}_{m}}{w_{c}{∆H}_{k}}\cdot 100\%$$

where

*S_K_*—degree of crystallinity, %;
${∆H}_{m}$—the enthalpy of fusion for the material examined, J/g;
$w_{c}$—the mass fraction of homopolymer added to the composite studied;
${∆H}_{k}$—the enthalpy of fusion for the entirely crystalline material for polyethylene—293, J/g [20,21,22,23].

#### 2.2.4. Microscopic Studies

A Keyence VHX-7000 (Keyence Ltd., Milton Keynes, UK) digital optical microscope and a scanning electron microscope JEOL JSM-6610 LV (Jeol, Akishima, Japan) with an EDS microanalyzer were used for observations of the specimen microstructure. Observations were carried out on metallographic samples. Prior to observations by scanning electron microscopy (SEM), all samples were sputter-coated with gold. These examinations were used to determine the microstructure of the materials studied and the analysis of the distribution and shape of the filler particles. Furthermore, the use of a chemical composition analyzer allowed for the analysis of the distribution of chemical elements in selected filler particles.

#### 2.2.5. Three-Point Bending Test

A bending strength test machine—Zwick/Roell Z100 (ZwickRoell Group, Wroclaw, Poland)—was used to test the bending strength. The three-point bending test was carried out in accordance with the current standard PN-79/C-89027 [24]. The speed of the test was 2 mm/min. The spacing between supports was 50 mm.

#### 2.2.6. Hardness

A type D hardness tester was used to test the hardness using the Shore method. The test consisted of measuring the resistance that the test material exhibits when plunging a cone of a fixed shape and dimensions into the material. The pressure on the test sample was 50 N. Hardness was expressed in Shore D units.

## 3. Results and Discussion

**Figure 2:**
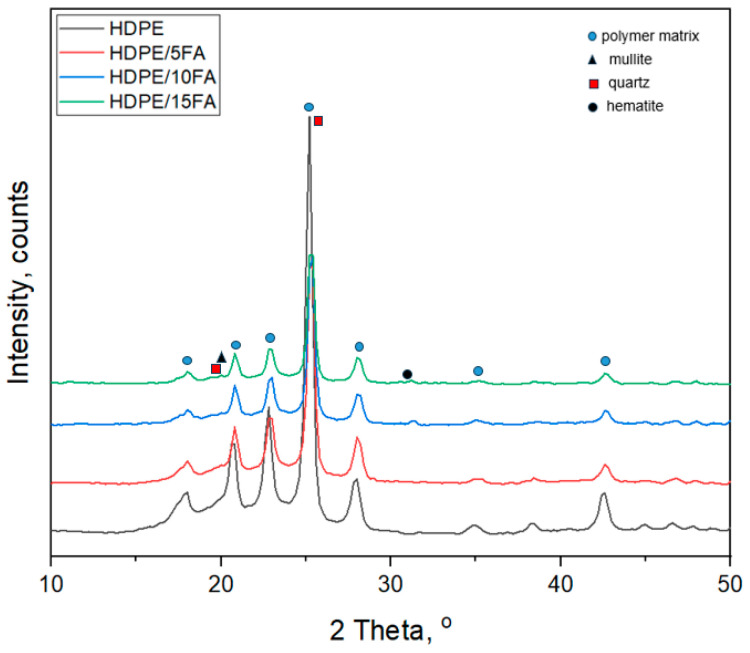
XRD diffraction of HDPE and HDPE/FA composites.

The results of the X-ray analysis of high-density polyethylene (HDPE) and high-density polyethylene composites with different fly ash contents (HDPE/FA) are presented in Figure 2 and Table 2.

XRD analysis demonstrated the presence of two crystalline phases of polyethylene in all the specimens tested: orthorhombic (ICDD00-060-0986) and monoclinic (ICDD 00-054-1981). According to the literature, the predominant crystalline phase in high-density polyethylene is the orthorhombic phase [25,26]. However, the polymorphism of this polymer is a frequently observed phenomenon when it is subjected to external forces and/or pressure that are present during extrusion or injection molding processes. These phases may differ in lamella thickness and orientation, spherulitic crystal structures, the presence of mesophases, or the elementary cell structure [27,28]. Analysis of the diffractogram of the tested materials revealed the presence of peaks at diffraction angles 2θ = 25.17° and 27.93°. Based on data from the literature [29] on the position of the characteristic diffraction reflections for HDPE, diffraction peaks were identified, originating from the orthorhombic HDPE corresponding to diffraction peaks from lattice planes (110) and (200). Furthermore, two weaker peaks were present at 2θ = 34.97° and 42.51°, which could be attributed to planes (210) and (020) of the polymer matrix, respectively. This is described in detail in the paper by Xiang et al. [30]. Diffraction peaks from the monoclinic HDPE occur at a diffraction angle of 2θ = 22.78° [31,32].

Crystallinity was calculated by determining the ratio of the area under the crystalline peaks to the total area under the diffraction curve for each material tested, as described in the study proposed by Costa et al. [33]. Analysis of the results of the calculations summarized in Table 2 found that the crystallinity decreased with increasing filler content. For pure HDPE, the crystallinity was 76.36%, while for the composites, it was HDPE/5FA (74.86%), HDPE/10FA (73.86%), and HDPE/15FA (72.23%), respectively. A higher filler content reduces nucleation and the possibility of further growth of the crystalline phase of the polymer matrix.

Based on full widths at half-maximum (FWHM), average crystallite sizes were calculated using the Scherrer Equation (2) in directions perpendicular to lattice planes with the following Miller indices: (110), (200)—diffraction peaks characteristic of the orthorhombic HDPE—and (010)—a diffraction peaks characteristic of the monoclinic HDPE.

(2) $$L_{c}=\frac{K\lambda }{\beta cos\theta },\;nm$$

where *K* is the Scherrer constant (typically taken as 0.9), *λ* is the X-ray wavelength used for the analysis (0.178901 nm), *β* is the full width at half-maximum (FWHM) of the diffraction peak in radians, and *θ* is the Bragg angle (diffraction angle) in radians.

The results of the crystallite size calculations presented in Table 2 indicate that the addition of fly ash slightly affects the size. As the filler content of the composites increases, the size of the crystallites in the polymer matrix decreases. Analysis of the diffractogram shown in Figure 1 reveals that as the fly ash content of the composites tested increases, the intensity of the individual diffraction peaks decreases, while the shape of the curves does not change significantly. Similar effects of fly ash in composites were documented in a study by Verma et al. [34]. According to the literature [35], the main crystalline phases occurring in fly ash from coal combustion are orthorhombic mullite, hexagonal quartz, and iron oxides: hematite and magnetite. This has been documented in several studies [1,2,36,37]. The addition of fly ash does not significantly alter the crystal structure of the polymer matrix [38]. In the diffractogram recorded, there were no clear peaks originating from the crystalline phases of the filler. The presence of phases derived from fly ash is most noticeable in the diffraction curve obtained for the HDPE/15FA composite. The low-intensity peaks recorded at diffraction angles, 2θ 19°, 26°, and 32°, are from mullite, quartz, and hematite, respectively.

**Figure 3:**
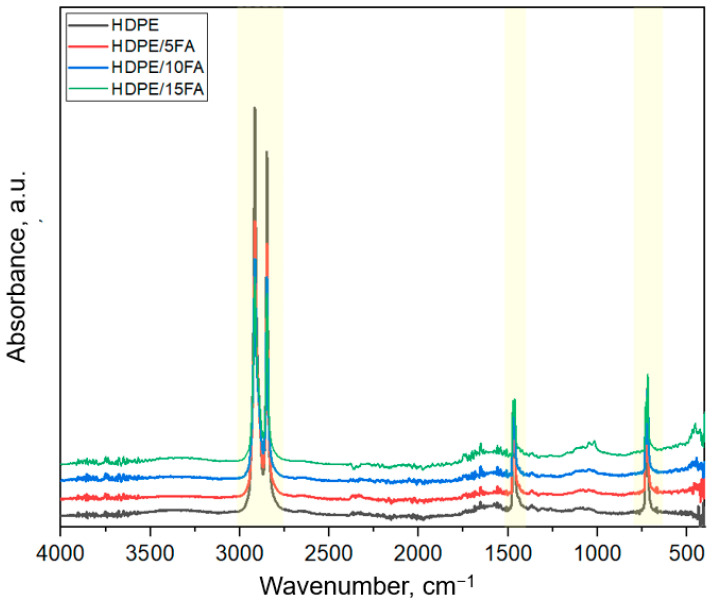
FTIR-ATR spectra of the studied materials.

Results of FTIR-ATR of high-density polyethylene and high-density polyethylene composites with different fly ash contents are shown in Figure 3.

In the IR spectrum, each absorbing group is assigned to an appropriate absorbing band with specific wavenumber values. Analysis of the FTIR-ATR spectra made it possible to identify characteristic absorbing groups present in the chemical structure of HDPE and HDPE matrix composites with different amounts of filler in the form of fly ash from coal combustion. Based on the available data from the literature [15,39,40,41], FTIR spectrum peaks of HDPE were assigned, as shown in Table 3.

FTIR-ATR infrared spectroscopy was used to estimate the crystalline phase content and analyze filler–polymer matrix interactions in HDPE/FA composites at different FA contents. The characteristic bands for polyethylene were observed in the spectra of all the materials studied.

**Figure 4:**
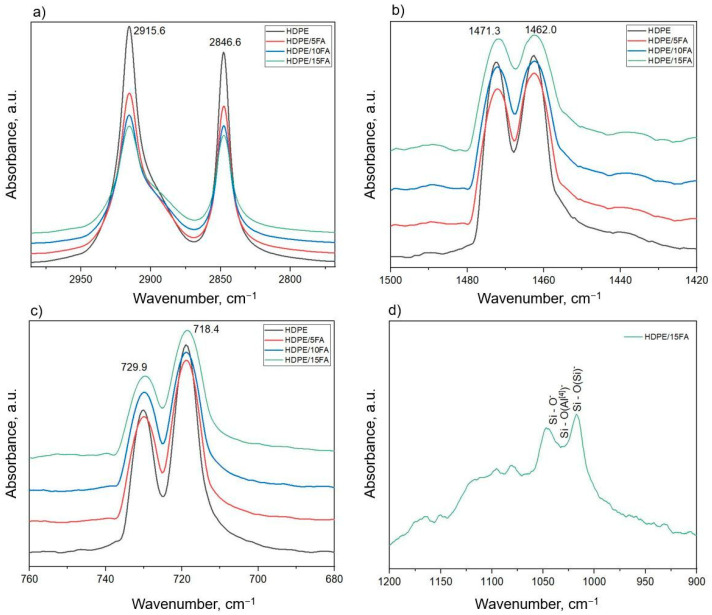
FTIR-ATR spectra for the HDPE and HDPE/FA composites (**a**) in the region 2800–2950 cm^−1^, (**b**) in the region 1420–1500 cm^−1^, (**c**) in the region 680–760 cm^−1^, and (**d**) in the region 900–1200 cm^−1^ for HDPE/15FA.

The strongest peaks occurring at wave numbers 2918 cm^−1^ and 2849 cm^−1^ (Figure 4a) were assigned to the asymmetric and symmetric stretching vibrations of methylene groups (-CH_2_-). The peaks occurring at wave numbers 1471 cm^−1^ and 1462 cm^−1^ were attributed to bending vibrations of the methylene groups (Figure 4b). Another strong band occurred at wave numbers 729 cm^−1^ and 719 cm^−1^. These peaks are characteristic of the rocking vibrations of the methylene groups of polyethylene (Figure 4c). The presence of fly ash in HDPE composites led to the appearance of a broad band in the FTIR-ATR spectrum, with the wave number ranging from approximately 900 to 1200 cm^−1^, which is associated with the high glass content (Figure 4d). This was most evident for a composite containing 15% FA. This band was formed from the superposition of three bands corresponding successively from the lowest wave number: asymmetric Si-O-stretching vibrations of broken oxygen bridges, asymmetric Si-O-stretching vibrations in the vicinity of tetrahedral aluminum, and asymmetric Si-O-stretching vibrations in the vicinity of silicon. For a wave number of approximately 460 cm^−1^ in the composite specimens (Figure 3), an O-Si-O bending vibration band maximum was observed, associated with the presence of silica glass and quartz [42,43,44].

FTIR-ATR can also provide additional information about the crystalline phase content of polymers, as some absorption peaks in the infrared spectrum of HDPE are sensitive to crystallinity. The crystalline forms of HDPE and the absorption of the amorphous phase can be determined by infrared active bending vibrations in the range 1471 to 1462 cm^−1^ and/or rocking vibrations in the spectral range 729 to 719 cm^−1^ [45]. The crystallinity was calculated based on the empirical relationships proposed by Zerbi et al. [46], where *X* is the percentage of the amorphous phase, and *I_α_* and *I_β_* are the infrared intensities in the spectral bands of the 1471 and 1462 cm^−1^ doublets or the 729 and 719 cm^−1^ bands. The 1471/729 cm^−1^ bands are crystalline bands, while the 1462/719 cm^−1^ bands are associated with the amorphous component. The ratio of band intensities in the spectrum of 100% crystalline PE is 1.233 [46].

(3) $$\%X=\frac{I_{\beta -\frac{I_{\alpha }}{1.233}}}{I_{\beta }-I_{\alpha }}\cdot 100$$

X_c_ crystallinity can be obtained in addition to the amorphous phase content (1-%X amorphous phase). The crystalline phase content was determined using the ratio of methylene rocking band intensities for the 729 and 719 cm^−1^ doublets. The degree of the crystalline phase content of HDPE and HDPE/FA composites is shown in Table 4.

After analysis of the results, it was observed that the crystallinity decreased from 74.8% for HDPE to 71.1% for the HDPE/15FA composite. This indicates that the degree of crystallinity decreased with increasing fly ash content in the composites, indicating that fly ash particles did not improve the crystallinity of the materials tested. The FTIR technique for determining the degree of crystallinity of HDPE with different fillers has been used in many studies. Tarani et al. [47] studied the effect of graphene nanoplatelets (GNPs), their content and size, on the crystallinity of HDPE/GNP composites. The crystalline phase content determined by FTIR spectroscopy for HDPE was approximately 76% (and a similar content was obtained in this study). A positive effect of GNP nanoparticles on crystallinity was observed at lower concentrations, while a higher nanofiller content resulted in the blocking of the molecular mobility of the HDPE polymer matrix, leading to lower crystallinity.

Stark and Matuana [48] used FTIR to analyze the change in chemical composition and crystallinity of HDPE and wood flour–HDPE matrix composites (WF/HDPE) under accelerated aging. They used the double peaks occurring at wave numbers 730 and 720 cm^−1^ to calculate the crystallinity. Longer specimen exposure times and the addition of wood flour led to a decrease in crystallinity. The FTIR technique for calculating the degree of crystallinity was also used in this study [46]. The authors studied medium-density polyethylene (MDPE) nanocomposites with different contents of carbon nanotubes (CNTs) and graphene nanoplatelets (GNPs). This study aimed to determine and compare the behavior of MDPE-based nanocomposites under proton irradiation. They used the intensity of the doublet bands occurring at wave numbers 729 cm^−1^ and 718 cm^−1^ to determine the degree of crystallinity. Analysis of the results revealed a slight decrease in crystallinity with an increasing GNP content, which could be attributed to the tendency of GNP to hinder the molecular mobility of the polymer matrix at relatively high concentrations (above 3–5%), thus limiting the growth of polyethylene crystallites. At concentrations of 5 wt%, MDPE/MWCNT (multi-walled carbon nanotube) specimens showed slightly higher X_c_ compared to MDPE/GNP nanocomposites because the GNP filler imposes more constraints around the polymer chains, causing more of the polymer chains to be trapped in the graphene lattice. All nanocomposites tested showed a decrease in crystallinity after proton irradiation. Furthermore, Kim and Lee [20] used double peaks occurring at wave numbers 1472 and 1462 cm^−1^ to calculate the changes in crystallinity of HDPE under the hydrogen treatment of specimens. After analysis of the results, they observed that the crystallinity decreased from 69% for HDPE before high-pressure hydrogen treatment to 63% after the treatment. This indicated a reduction in the degree of crystallinity of the HDPE specimens as a result of the hydrogen treatment.

**Figure 5:**
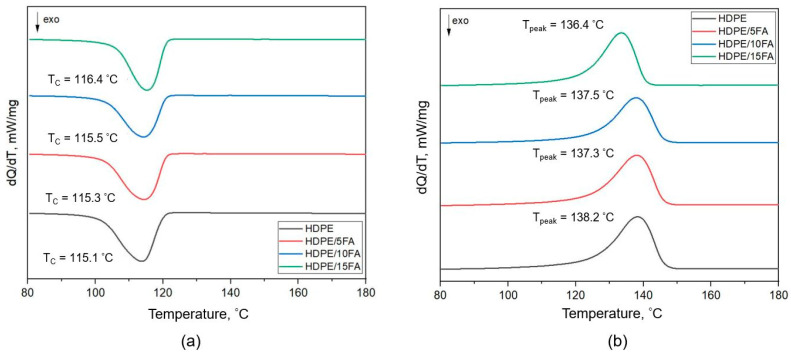
Thermograms of DSC: (**a**) cooling and (**b**) heating scans for the HDPE and HDPE/FA composites.

Differential scanning calorimetry (DSC) was another research technique used to analyze the changes in the crystalline structure (degree of crystallinity) and thermal properties of the materials induced by the fly ash filler. The results of the analysis using differential scanning calorimetry are presented in Figure 5 and Table 5.

The shape of the DSC curves does not indicate the presence of more than one polyethylene crystalline fraction in the materials tested. An analysis of the results shown in Table 5 reveals small changes in the values of the crystallization temperature T_C_ and melting temperature T_peak_. Only in the case of the HDPE/15FA composite can an increase in the crystallization temperature of 1 °C be observed while the melting point value decreases. A similar filler effect was found in medium-density polyethylene (MDPE) nanocomposites, with carbon nanotubes (CNTs) and graphene GNP nanoplatelets used as fillers [49].

A reduction in the melting point indicated that the crystallization process occurred faster, and the crystals formed were smaller [50]. This was confirmed by the results of the XRD study (Table 2). The crystallite size in the polymer matrix for the composite containing 15% FA was lower compared to the crystallite size of pure HDPE. The reduction in crystallinity for the composites could be attributed to the inhibition of tight chain packing due to increased interactions between the polymer matrix and fly ash particles. The same effect of the filler on the HDPE matrix was observed by Divya et al. [51]. They studied the effect of the addition of cenospheres and MWCNTs (multi-walled carbon nanotubes) on the mechanical, thermal, and fire resistance properties of HDPE matrix composites. These authors found that the percentage of crystallinity determined by the DSC technique decreased by 13.6% when 20% cenospheres were added to the polymer matrix.

**Figure 6:**
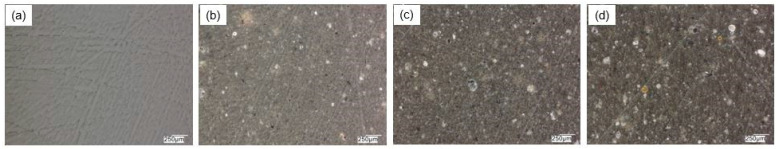
Microstructure of high-density polyethylene (**a**) and high-density polyethylene composites with varying fly ash content at 100× magnification: (**b**) HDPE/5FA, (**c**) HDPE/10FA, and (**d**) HDPE/15FA.

Structural examinations of HDPE composites with different fly ash contents were performed using a digital optical microscope. Observations were carried out on metallographic sections using 100× magnification. The microstructure photographs obtained for the materials studied are presented in Figure 6.

Fly ash particles from coal combustion are evenly distributed in the polymer matrix. The shape of the particles is spherical, and their sizes are highly variable. Most particles have dimensions of a few to tens of micrometers. The color of the particles can be grey, grey-brown, light brown, or black. Some authors believe [37,52] that the different colors are due to the different proportions of SiO_2_ and Al_2_O_3_ but can also result from elements such as iron, potassium, sodium, magnesium, and titanium. The dark color of the fly ash is caused by parts of unburned carbon or magnetite grains. The brown color is due to the presence of hematite grains.

**Figure 7:**
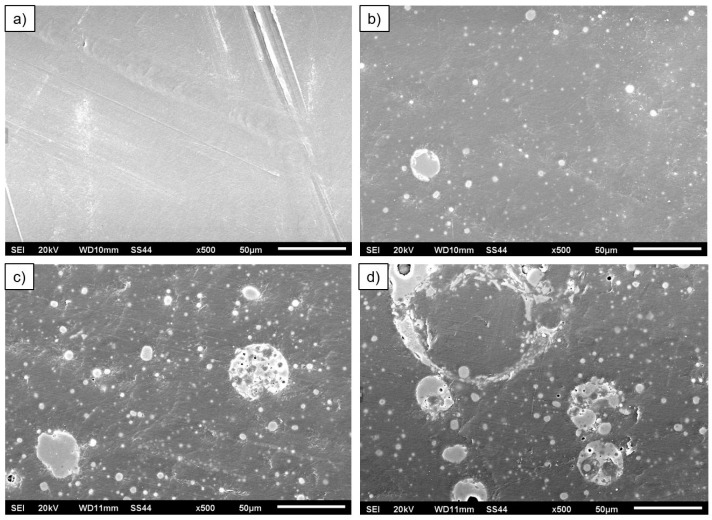
SEM microstructure of the materials studied: (**a**) HDPE, (**b**) HDPE/5FA, (**c**) HDPE/10FA, and (**d**) HDPE/15FA.

Observations of the structure of the materials studied were also performed using a scanning microscope. The microstructure photographs obtained for the materials studied are presented in Figure 7.

Morphologically, fly ash grains are highly variable. Fly ash consists mainly of spheroidal and spherical grains. The most abundant ash fraction is very fine, with spherical and oval-shaped solid grains. Vitreous spheres filled with gases such as H_2_, N_2_, CO, H_2_O, Ar, CO_2_, known as cenospheres, most often represent the coarser fractions of fly ash, sometimes exceeding 100 μm in diameter. Spheres can also occur as multispheres, known as plerospheres, which are filled with smaller spherical grains [2]. Irregular grains with a rough or spongy surface can also be observed on the presented microstructures. According to the literature [36,53], these can be crumbs of unburned coal and quartz grains (larger grains) or minerals whose structure was destroyed during thermal processes, but the grains were not melted. The finest fractions generally constitute graphite dust. 

**Figure 8:**
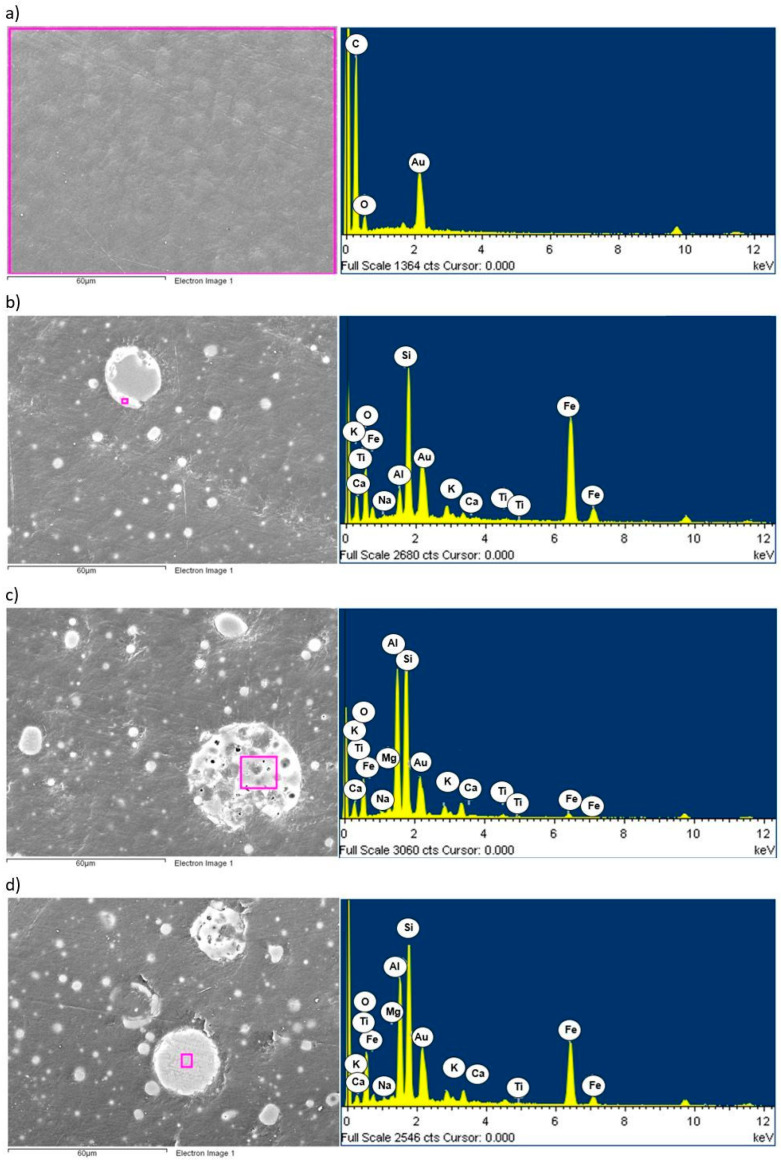
Area and spectrum of EDX analysis for HDPE. (**a**) HDPE, (**b**) HDPE/5FA, (**c**) HDPE/10FA, (**d**) HDPE/15FA.

The chemical composition was analyzed using the EDS attachment of a JEOl scanning microscope (JEOL JSM-6610 LV, Jeol, Akishima, Japan). The microanalysis of the chemical composition of the filler particles was performed in selected areas of the composite specimens. The chemical composition of the polymer matrix (HDPE) was also analyzed. The distributions of elements in the tested materials are shown in Figure 8.

The microstructure of the tested composites (Figure 8) showed spherical and irregularly rounded fly ash particles made up of glaze. Si, Al, and K were present in the chemical composition of the glaze of the fly ash studied. Some of the particles also contained Fe, indicating the presence of an overgrown iron phase in the form of hematite or magnetite. Crystalline and dendritic spherical grains were composed of Fe oxides. These phases, in the form of independent grains, occurred sparsely in the fly ash, whereas they relatively often overgrew the glaze and filled the cenospheres [54]. The spherical particle seen in Figure 8d is characterized by a dendritic–skeletal structure, characteristic of magnetite, which is connected by aluminosilicate glass [36]. However, the occurrence of this phase was not confirmed by X-ray examinations. This may be due to its low percentage (below the detectability of the X-ray diffraction method used) or a low degree of crystallization [36,52].

**Figure 9:**
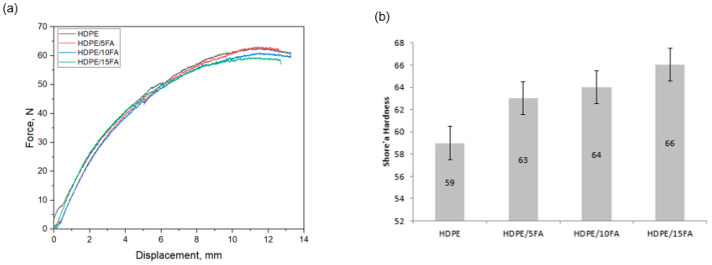
The mechanical properties of the materials tested: (**a**) three-point bending test; (**b**) Shore hardness measurements.

The effect of the addition of coal combustion fly ash on the mechanical properties of the composites tested was also analyzed. A three-point bending test and Shore hardness measurements were carried out. The results are shown in Figure 9.

Analyzing the recorded bending curves shown in Figure 9a, no significant differences were observed. Among the tested composites, the maximum load transferred (25 MPa) was demonstrated by the composite sample with the addition of 5% coal ash, with a deflection angle of α = 70°. All specimens showed high ductility and did not fracture during the test. Analyzing the results of the hardness measurement (Figure 9b), it can be seen that as the fly ash content increased, the hardness increased. High-density polyethylene has a hardness of about 58°ShD. The addition of the filler causes a slight increase in hardness. Polyethylene with a 15% filler content has the highest hardness of about 66° ShD.

## 4. Conclusions

XRD analysis showed the presence of two crystalline phases of high-density polyethylene in all the samples tested: an orthorhombic and monoclinic phase. The addition of fly ash did not significantly change the crystallography of the polymer matrix. The presence of fly ash-derived phases was most noticeable in the diffraction curve obtained for the HDPE/15FA composite.

FTIR-ATR infrared spectroscopy was used to estimate the crystalline phase content and analyze filler–polymer matrix interactions in HDPE/FA composites at different FA contents. In the FTIR-ATR spectrum, the presence of fly ash in HDPE composites becomes apparent in the form of a broad band in the wavelength number range of 900–1200 cm^−1^, associated with its chemical structure, which is particularly evident with a higher content of the filler in the composite. 

The addition of the filler lowers the melting point, the crystallization process occurs faster, and the crystals formed are smaller. The introduction of fly ash into the polymer matrix results in a lower degree of crystallinity. High-density polyethylene composites filled with more fly ash particles have a lower degree of crystallinity than unmodified HDPE. It is reasonable to assume that the filler particles impede the diffusion of macromolecule segments into the growing crystallites, resulting in the formation of a smaller volume of the crystalline phase. 

A uniform distribution of fly ash particles in the polymer matrix and the absence of conglomerates were observed. The fly ash grains observed on the microstructures of the studied composites were spherical in shape, and their sizes were highly variable. As a result of changes in the structure, including the content of the crystalline phase, the functional properties changed, there was an increase in hardness depending on the filler content in the composite relative to the starting polymer, and to a small extent, there was flexural strength in the case of the HDPE/5FA composite. Such favorable changes and the possibility of obtaining products from waste and recycled materials provide the possibility of using composites in various industries. The developed method of manufacturing composite materials provides an easy and inexpensive way to obtain a product with many geometric parameters, suitable for direct use as components of machinery and equipment in the engineering industry, automotive industry, and especially for the manufacture of components of household appliances, sanitary, electrical appliances, in industrial applications and equipment used in various industries and intended for various types of applications.

## Figures and Tables

**Table 1:** Determination of test samples.

Specimen	HDPE	HDPE/5FA	HDPE/10FA	HDPE/15FA
Fly ash from hard coal combustion, wt%	-	5	10	15

**Table 2:** Summary of the diffraction peak parameters of the tested materials.

Material	Pic 2θ, °	Intensity, Counts	FWHM	Crystallinity, %	Size of the Crystalline Domains, nm
HDPE	25.17	13,000.0	0.49	76.36	19.50
	27.93	1735.9	0.64		15.02
	22.78	3973.8	0.65		14.64
HDPE/5FA	25.20	6775.1	0.50	74.86	19.12
	28.04	1486.8	0.65		14.79
	22.91	2085.9	0.68		14.00
HDPE/10FA	25.26	5224.0	0.51	73.86	18.74
	28.06	962.3	0.66		14.57
	22.91	1486.77	0.69		13.79
HDPE/15FA	25.27	2592.3	0.52	72.23	18.38
	28.06	634.6	0.67		14.35
	22.90	1109.01	0.70		13.60

**Table 3:** Characterization of the FTIR-ATR spectrum of the tested materials.

Peak Number	Band Wave Number, cm^−1^	Absorbing Bands	Type of Vibrations	Intensity	Phase Type
1	2918	CH_2_	asymmetric stretching	Strong	
2	2849	CH_2_	symmetric stretching	Strong	
3	1471	CH_2_	C-H bending vibrations	Strong	crystalline
4	1462	CH_2_	C-H bending vibrations	Strong	amorphous
5	729	CH_2_	C-H rocking vibrations	Medium	crystalline
6	719	CH_2_	C-H rocking vibrations	Medium	amorphous

**Table 4:** Crystallinity determined by FTIR-ATR method.

Material	Crystallinity X_c_, %
HDPE	74.8
HDPE/5FA	74.4
HDPE/10FA	73.2
HDPE/15FA	71.1

**Table 5:** DSC results.

	Peak Temperature, T_C_, °C	Crystallinity X_c_, %	Peak Temperature T Peak, °C
HDPE	115.1	73.2	138.2
HDPE/5FA	115.3	69.0	137.3
HDPE/10FA	115.5	65.8	137.5
HDPE/15FA	116.4	60.2	136.4

## Data Availability

The original contributions presented in the study are included in the article, further inquiries can be directed to the corresponding author.
